# Spatial-Niche Perspective on the Heterogeneity and Functional Reprogramming of Tumor-Associated Macrophages in Digestive System Tumors

**DOI:** 10.3390/cells15131198

**Published:** 2026-07-01

**Authors:** Jingcheng Zhang, Yi Huang, Mingsi Zhang, Jiaheng Lou, Shuo Zhang, Sicheng Zhao, Zhiyuan Song, Kaiyuan Zhang, Tao Jiang, Guangji Zhang

**Affiliations:** 1 School of Basic Medical Sciences, Zhejiang Chinese Medical University, Hangzhou 310053, Chinaljh990704@163.com (J.L.); zs15156020951@163.com (S.Z.); xczyzsc@163.com (S.Z.); 16639178019@163.com (Z.S.); 202111120611045@zcmu.edu.cn (K.Z.); 2 Zhejiang Key Laboratory of Blood-Stasis-Toxin Syndrome, Zhejiang Chinese Medical University, Hangzhou 310053, China; 3 Traditional Chinese Medicine “Preventing Disease” Wisdom Health Project Research Center of Zhejiang, Hangzhou 310053, China

**Keywords:** tumor-associated macrophages, digestive system tumors, spatial niches, tumor microenvironment, macrophage heterogeneity, spatial transcriptomics, functional reprogramming, immunotherapy

## Abstract

**Highlights:**

**What are the main findings?**

- A spatial niche-based framework is proposed to interpret tumor-associated macrophage heterogeneity in digestive system tumors.
- Six recurrent spatial niches are integrated with functional axes linking microenvironmental cues to TAM programs and outputs.

**What are the implications of the main findings?**

- Spatial context provides a refined perspective beyond conventional TAM subtype classification.
- Niche-specific TAM programs offer potential targets for spatially guided immunotherapy.

**Abstract:**

Tumor-associated macrophages (TAMs) are among the most important myeloid cell populations in the tumor microenvironment of digestive system tumors and are characterized by marked plasticity, heterogeneity, and context dependence. This review focuses on gastric, colorectal, liver, and pancreatic cancers as representative digestive system solid tumors in which TAM spatial organization has been increasingly characterized by single-cell and spatial omics studies. Traditional M1/M2 polarization or fixed subtype-based classification is insufficient to capture the continuous state transitions of TAMs across tumor types, disease stages, and tissue regions. Recent evidence suggests that TAM heterogeneity reflects dynamic functional states shaped within distinct spatial niches by local oxygen supply, metabolic stress, stromal architecture, vascular status, and interactions with neighboring cells. From a spatial-niche perspective, this review synthesizes current evidence on TAM distribution patterns, phenotypic changes, and functional biases across six recurrent spatial contexts: the hypoxic core, invasive front, fibrotic septa, perivascular regions, tertiary lymphoid structure (TLS)-adjacent regions, and necrotic borders. By linking these niches with cross-niche functional axes and evidence-supported molecular programs, we provide a structured niche-to-function framework for comparing TAM spatial heterogeneity and its major functional dimensions, including metabolic adaptation, tissue remodeling, and immune-inflammatory regulation. This context-sensitive framework may help guide future studies of niche-specific TAM reprogramming and rational combinations with immunotherapy and other treatment strategies.

## 1. Introduction

Digestive system tumors comprise a broad group of malignancies, among which gastric cancer, colorectal cancer, liver cancer, and pancreatic cancer are major representative solid tumors that together account for approximately 2.8 million cancer-related deaths worldwide, according to GLOBOCAN 2022 estimates [1,2]. In addition to marked genomic diversity, these malignancies arise and evolve within a highly complex tumor microenvironment (TME), which profoundly influences tumor progression and therapeutic response [3,4,5]. The TME comprises multiple immune, stromal, vascular, and metabolic components. These components are not uniformly distributed, but instead form spatially distinct local environments within tumor tissues, thereby jointly shaping tumor initiation, invasion, and metastasis [6,7,8]. Cues such as hypoxia, nutritional and metabolic gradients, cytokine distribution, mechanical stress, and extracellular matrix (ECM) architecture further organize these local environments into distinct spatial niches and exert differential effects on resident and infiltrating immune cells [9,10,11].

Tumor-associated macrophages (TAMs) are among the most abundant innate immune cells in digestive system tumors and exert broad effects on tumor–immune dynamics and local tissue architecture [12,13,14,15]. Increasing evidence indicates that TAMs are not predefined static subsets, but rather dynamic cellular states continuously shaped by local factors such as hypoxia, metabolic stress, cytokine signaling, and ECM mechanical properties [16,17,18]. More importantly, TAMs do not merely passively respond to niche-derived signals; instead, they can actively remodel their surrounding microenvironment by producing pro-angiogenic factors, immunoregulatory mediators, matrix-modifying enzymes, and metabolic regulatory molecules, thereby establishing sustained feedback with local niches [19,20]. This bidirectional interaction supports the use of spatially defined local contexts to complement conventional polarization frameworks or static subset classifications when interpreting TAM heterogeneity.

Although single-cell and spatial omics studies have substantially advanced our understanding of TAM heterogeneity and recurrent TAM functional programs in digestive system tumors have gradually been described, their spatial organization and the mechanisms linking niche-specific pressures to functional outputs remain insufficiently defined [21,22,23]. For example, enrichment of TAMs in the hypoxic core, invasive front, tertiary lymphoid structures, and fibrotic septa has been repeatedly reported, yet a unified framework is still lacking to explain how spatial factors shape TAM state shifts and functional biases, and how these changes further influence immunosuppression, therapeutic resistance, and potential translational vulnerabilities [24]. This gap limits our ability to understand TAM biology through spatial information and to develop more refined intervention strategies.

Digestive system tumors provide a particularly relevant setting for a spatial-niche perspective because they are shaped by distinct tissue and immune contexts, including the mucosal barrier, gut-derived antigens and microbial exposure, portal venous inflow, hepatic immune tolerance, and fibrotic remodeling [25,26,27,28,29,30]. This review focuses on gastric cancer, colorectal cancer, liver cancer, and pancreatic cancer as representative digestive system solid tumors, where current single-cell and spatial omics studies have increasingly described TAM distribution, niche-associated cellular neighborhoods, and local functional programs [31,32,33,34,35]. Building on these observations, this narrative and conceptual synthesis integrates recurrent niche contexts, TAM-associated molecular features, dominant biological roles, and evidence sources into a comparative niche-to-function framework that provides a spatially organized basis for studying TAM heterogeneity. This six-niche, three-axis framework supports structured comparison of TAM-associated programs across spatial settings and helps link spatial organization with functional interpretation, thereby providing a practical foundation for future spatially resolved studies of macrophage organization, niche-associated TAM functions, and candidate macrophage-targeted strategies that take local microenvironmental features into account [36,37].

The literature discussed in this narrative review was identified through searches of PubMed, Web of Science, and Google Scholar from database inception to June 2026, using combinations of terms related to tumor-associated macrophages, spatial niches, spatial transcriptomics, single-cell RNA sequencing, digestive system tumors, gastric cancer, colorectal cancer, liver cancer, pancreatic cancer, hypoxia, fibrosis, invasive front, perivascular regions, tertiary lymphoid structures, and necrosis. We prioritized single-cell, spatial omics, multiplex imaging, and mechanistic studies in digestive system tumors, while also including selected broader tumor, fibrosis, biomaterial, experimental, and high-quality review studies when they provided mechanistic or conceptual support for niche-associated TAM programs.

## 2. Major Spatial Niches in Digestive System Tumors

In digestive system tumors, spatial heterogeneity can be further manifested as several spatial microenvironmental compartments with relatively stable local environmental features [38,39]. These compartments are not merely morphological partitions, but are better understood as regional microenvironmental units jointly defined by local oxygen supply and metabolic status, stromal architecture, vascular perfusion, cellular composition, and spatial proximity relationships [40,41]. In recent years, with advances in spatial transcriptomics, multiplexed imaging, and digital pathology, several recurrent spatial compartments within digestive system tumors have gradually been described, enabling the spatial organization of the tumor microenvironment to be identified at higher resolution [38,42,43]. Based on these observations, this review summarizes six major spatial niches that are particularly relevant to TAM organization: the hypoxic core, invasive front, fibrotic septa, perivascular regions, tertiary lymphoid structure (TLS)-adjacent regions, and necrotic borders. Their defining microenvironmental features and representative organ contexts are summarized in Table 1. It should be emphasized that these niches are neither mutually exclusive nor an exhaustive classification within a single partitioning dimension. Rather, they represent recurrent spatial contexts in digestive system tumors that are useful for comparing TAM spatial heterogeneity. In actual tissues, these niches may partially overlap. They may also reflect different local features, including metabolic or perfusion status, tissue interfaces, stromal architecture, vascular proximity, immune organization, and injury-associated transitions.

### 2.1. Hypoxic Core

The hypoxic core is a typical high-metabolic-stress niche in digestive system tumors and is usually located in deep tumor regions distant from effective perfusion [44]. As tumor cells continue to proliferate, local demand for oxygen and nutrients increases, whereas abnormal vascular architecture, poor perfusion, and reduced efficiency of material exchange limit supply capacity. As a result, this region often develops environmental features such as persistent hypoxia, lactate accumulation, acidosis, nutrient deprivation, and metabolic waste buildup [45,46,47,48]. Under this sustained demand–supply imbalance, the hypoxic core represents a metabolically constrained tumor niche [49,50,51].

In digestive system tumors, such regions can be observed in rapidly growing solid lesions with prominent vascular abnormalities [52,53,54]. Across different organ contexts, this niche is defined primarily by constrained perfusion, restricted material exchange, impaired oxygen supply, and limited local immune activity [55,56,57].

### 2.2. Invasive Front

The invasive front is a dynamic interface formed as tumor cells expand into surrounding tissues and come into direct contact with the stroma, and is usually located at the boundary between the tumor parenchyma and host tissue [58]. Compared with deep tumor regions, this area is more directly exposed to continuous tumor–stroma interactions and therefore remains in a state of active structural remodeling [59]. Its major features include disruption and reorganization of tissue architecture, extracellular matrix remodeling, cancer-associated fibroblast (CAF) activation, increased cellular migratory activity, and reinforced epithelial–stromal interactions [60]. Therefore, the invasive front represents an active region of local structural remodeling and cellular interaction during tumor expansion.

Across digestive system tumors, the specific morphology of the invasive front may vary with the local tissue architecture, including mucosal, glandular, stromal, or parenchymal boundaries. Its shared defining feature is the local convergence of boundary disruption, matrix remodeling, inflammatory signals, and growth factor cues during tumor expansion [38,61,62].

### 2.3. Fibrotic Septa

Fibrotic septa represent a structural niche dominated by dense stromal architecture in digestive system tumors. Their major features include increased collagen deposition, extracellular matrix crosslinking, elevated stromal density, increased tissue stiffness, and reduced local permeability [63,64,65]. Morphologically, these regions often appear as cord-like, septal, or lamellar fibrotic structures that separate tumor compartments and constrain cellular migration, local perfusion, and drug diffusion [66,67,68]. Although their prominence varies across digestive system tumors, fibrotic septa are mainly defined by structural barrier formation and mechanical constraint within matrix-rich tumor regions [13,69].

### 2.4. Perivascular Regions

Perivascular regions are interface niches distributed around tumor-associated blood vessels and endothelial structures. They are located in the transitional zone between the vascular lumen, vessel wall, and adjacent tumor parenchyma, where oxygen, nutrients, circulating factors, therapeutic agents, and immune cells can enter or interact with tumor tissue [70,71,72]. Compared with normal tissues, tumor-associated vasculature often exhibits structural disorganization, incomplete vessel walls, perfusion fluctuations, increased permeability, altered local fluid pressure, and persistent vascular remodeling [73,74,75]. Across digestive system tumors, perivascular regions are therefore defined primarily as vascular-interface niches shaped by endothelial proximity, unstable perfusion, vascular permeability, and exchange between circulation and tumor tissue [76].

### 2.5. TLS-Adjacent Regions

Tertiary lymphoid structure-adjacent regions represent spatial niches closely associated with local adaptive immune responses in digestive system tumors [77,78]. As ectopically formed lymphoid structures, TLSs can organize immune components such as T cells, B cells, and dendritic cells within tumor tissues, providing a structural basis for local antigen presentation and lymphocyte interactions [79,80]. As a result, their surrounding regions typically show higher lymphocyte density, broader antigen exposure, and more active immune cell communication [81,82,83]. Therefore, this region can be understood as a microenvironmental compartment characterized by immune-cell aggregation and active local immune communication [84,85].

TLS-adjacent regions do not necessarily correspond to a uniformly antitumor immune state. Existing studies suggest that immune activation and immune restriction can coexist within and around TLSs, and this spatial heterogeneity is associated with differences in local cellular composition, cytokine balance, and inhibitory molecule distribution [41,84]. Across digestive system tumors, the shared defining feature of TLS-adjacent regions is dense spatial organization of immune interactions, while their specific functional states may vary with the local immune and inflammatory background [85,86].

### 2.6. Necrotic Borders

Necrotic borders are spatial niches located between necrotic foci and viable tumor tissue, with prominent injury-response features in digestive system tumors [87]. These regions are characterized by collapsed oxygen supply, metabolic disruption, exposure to damage-associated molecular patterns released by necrotic cells, oxidative stress, and inflammatory mediators [88,89]. As a result, their local environment combines tissue injury, cellular debris accumulation, inflammatory activation, and phagocytic clearance programs [90,91].

Compared with other niches, necrotic borders are defined by diverse and changing local stimuli. Necrosis-associated signals confer acute injury-like features, whereas the surrounding chronic tumor microenvironment continuously reshapes these responses [92,93]. Overall, necrotic borders represent stressed transition zones formed by the interaction among necrosis, inflammation, residual viable tumor tissue, and local immune regulation [94,95,96].

### 2.7. Summary

The hypoxic core, invasive front, fibrotic septa, perivascular regions, TLS-adjacent regions, and necrotic borders summarize several key spatial contexts frequently observed in digestive system tumors. These contexts correspond to local microenvironmental states dominated by metabolic stress, tissue-interface remodeling, stromal architecture, vascular exchange, immune organization, and injury responses, respectively. They are not mutually exclusive, nor do they constitute an exhaustive classification of tumor spatial states. Rather, they provide a comparative framework for organizing recurrent histological and microenvironmental features across digestive system tumors and for analyzing niche-associated TAM states and functions.

## 3. TAM Features and Functions Across Spatial Niches

**Figure 1:**
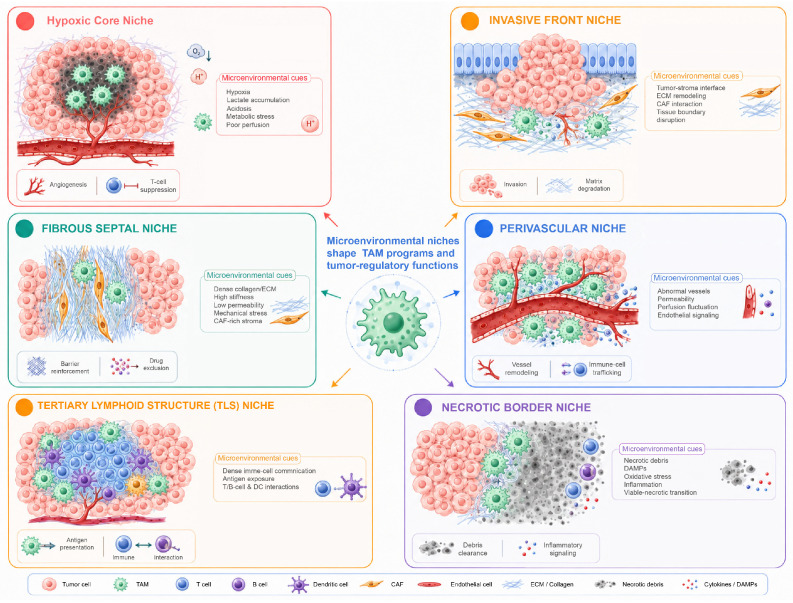
Niche-specific TAM-related microenvironmental cues and functional outputs across major spatial niches in digestive system tumors.

Oxygen supply and metabolic status, stromal conditions, and vascular-related environments differ across spatial niches, and these local differences can continuously shape the state features and functional tendencies of TAMs, leading them to exhibit region-adapted phenotypic heterogeneity within tumor tissues [18,97,98]. Therefore, the spatial niches described above not only provide a structural framework for understanding the organization of the tumor microenvironment in digestive system tumors, but also offer a specific context for analyzing the spatially dependent heterogeneity of TAMs. As illustrated in Figure 1, each recurrent niche can be described by a characteristic combination of local microenvironmental cues and TAM-associated functional outputs. On this basis, this review separately examines TAM features and functions in the hypoxic core, invasive front, fibrotic septa, perivascular regions, TLS-adjacent regions, and necrotic borders. Here, “TAM features” mainly refer to molecular expression tendencies, spatial proximity relationships, and histological states within specific spatial contexts, whereas “TAM functions” refer to the modes of action corresponding to these features within local microenvironments. Accordingly, the following subsections integrate spatial distribution, molecular programs, and local histological context to define niche-associated TAM states and their dominant functional orientations.

This schematic summarizes six recurrent spatial niches relevant to tumor-associated macrophage (TAM) heterogeneity in digestive system tumors: the hypoxic core, invasive front, fibrotic septal, perivascular, tertiary lymphoid structure-adjacent (TLS-adjacent), and necrotic border niches. Each panel highlights representative local microenvironmental cues and major TAM-associated functional outputs within the corresponding niche. These include hypoxia-, lactate-, and poor-perfusion-related cues in the hypoxic core; tumor–stroma interface remodeling at the invasive front; dense collagen/ECM, high stiffness, and low permeability in fibrotic septa; abnormal vessels, perfusion fluctuation, and endothelial signaling in perivascular regions; dense immune-cell communication and antigen exposure around TLSs; and necrotic debris, DAMPs, oxidative stress, and inflammation at necrotic borders. The bottom legend defines the main cell types, structural components, and signals shown in the figure, including tumor cells, TAMs, T cells, B cells, dendritic cells, cancer-associated fibroblasts (CAFs), endothelial cells, extracellular matrix (ECM)/collagen, necrotic debris, and cytokines/damage-associated molecular patterns (DAMPs). Abbreviations: TAMs, tumor-associated macrophages; CAFs, cancer-associated fibroblasts; ECM, extracellular matrix; TLS, tertiary lymphoid structure; DAMPs, damage-associated molecular patterns; DCs, dendritic cells.

Together, these six niches expose TAMs to distinct local cues that shape representative molecular features and functional outputs. Figure 1 summarizes the major niche-specific TAM features discussed in Section 3.

### 3.1. TAM Features and Functions in the Hypoxic Core

In the hypoxic core, persistent oxygen deprivation, lactate accumulation, acidosis, and restricted nutrient exchange collectively shape TAMs toward metabolically adapted states [99,100,101]. At the molecular level, this adaptation is reflected by hypoxia-responsive and glycolysis-related molecules such as HIF1A, SLC2A1, and LDHA [95,102,103], together with angiogenesis- or recruitment-associated features represented by VEGFA and CXCR4 [95,102,103,104]. In some contexts, SPP1-related remodeling features may further support local vascular and matrix remodeling [95], whereas ARG1, CD163, and MRC1 represent immunosuppression-related expression tendencies [101,105,106]. Consistent with this interpretation, lactate–MCT–HIF signaling can promote macrophage polarization and immunosuppressive reprogramming [101,106], and TAMs in this niche are often associated with restricted effector T-cell function, enhanced local immunosuppression, and weakened antitumor responses [107].

The microenvironmental basis of this hypoxia-adapted TAM state differs across digestive system tumors, but most routes point to the shared pressure of insufficient local oxygen supply and restricted material exchange. In pancreatic cancer, this state is commonly embedded in dense stromal compression and persistent poor perfusion [103,105,108]; in liver cancer, it is more often related to oxygen supply imbalance caused by abnormal vascular architecture and rapid tumor growth, with spatial evidence linking hypoxic regions to SPP1+ macrophage accumulation and poor prognosis [95,102]; and in some gastric cancers, similar states are frequently associated with insufficient perfusion in deep tumor regions and restricted local material exchange [31,109]. Therefore, hypoxic cores can drive TAMs toward metabolically adapted states across different organs, but differences in the combination of local pressures, including stromal compression, vascular abnormality, insufficient perfusion, and restricted material exchange, may still shift the relative emphasis of their pro-angiogenic, remodeling-related, and immunosuppressive outputs [52,53,54].

Taken together, metabolic adaptation of TAMs in the hypoxic core is associated with both tolerance to hypoxic and acidic stress and participation in aberrant vascular remodeling and local immunosuppression [99,100,101,102,104,106,107].

### 3.2. TAM Features and Functions at the Invasive Front

At the invasive front, TAMs are mainly embedded within a continuously remodeled tumor–stroma interface. This region brings TAMs into close proximity with invasive tumor cells, CAFs, disrupted tissue boundaries, and a reorganizing extracellular matrix, thereby involving them in interface remodeling and stromal signal exchange [61,110]. In this context, TAM-associated molecular features often include matrix degradation and remodeling molecules, such as MMP9, MMP14, CTSB, and CTSD [111,112], together with stromal signaling or remodeling-related factors such as TGFB1 and SPP1 [111,113,114]. Features related to adhesion and matrix interaction, including FN1 and integrin-family molecules, further indicate a possible association with cell adhesion, matrix reconstruction, and structural adjustment at the tumor–stroma interface [111,112]. In addition, TAMs in this region may also undergo a degree of metabolic adjustment to sustain their activation and responsiveness within the dynamic interface environment [60]. Together, these molecular programs point to the involvement of invasive-front TAMs in matrix degradation, stromal remodeling, boundary adaptation, and local immune regulation.

The invasive front has different tissue boundaries across digestive system tumors, but its shared feature is continuous interface remodeling between tumor cells and surrounding tissues during tumor expansion. In gastric cancer and colorectal cancer, this process is more directly manifested as mucosal–basement membrane breach, glandular boundary disruption, and tumor expansion into surrounding stromal regions [61,115,116]. In liver cancer, the relevant TAMs more often participate in remodeling at the interface between tumor cells, hepatic sinusoids, and adjacent liver parenchyma [65,110,117]. In colorectal cancer, spatial and single-cell studies also support the presence of SPP1+ macrophage-related remodeling features and immune-regulatory interactions around invasive or tumor–stroma interface regions [114,116].

Taken together, invasive-front TAMs can be understood as cellular states involved in tumor–stroma interface regulation. Their matrix-degrading and adhesion-related programs may facilitate extracellular matrix turnover, tissue boundary adjustment, and tumor–stroma crosstalk, whereas TGFB1- or SPP1-associated signaling may be linked to local immune restriction and the formation of remodeling-supportive microenvironments [61,113,114].

### 3.3. TAM Features and Functions in Fibrotic Septa

The prominence of fibrotic septa differs across digestive system tumors. In pancreatic cancer, desmoplasia is a major feature of the tumor microenvironment, and TAM–CAF interactions and stromal reinforcement are usually more pronounced; dense stroma and restricted perfusion can further limit nutrient exchange and metabolite diffusion, placing TAMs in a low-exchange and low-permeability local environment [108,118,119]. In liver cancer, these TAMs are more readily embedded in stromal remodeling programs that extend from chronic liver disease and hepatic fibrosis; some gastric cancers may also show marked stromal septa, although these structures usually do not constitute a dominant niche to the same extent as in pancreatic cancer [13,120]. Therefore, unlike the hypoxic core or invasive front, fibrotic septa first represent a spatial niche dominated by tissue architecture and stromal-barrier formation.

In this niche, TAMs are mainly located within a barrier-like stromal architecture characterized by dense ECM deposition, collagen crosslinking, increased matrix stiffness, and reduced permeability. This environment places TAMs in close spatial proximity to CAFs and matrix-rich regions, situating their states within a fibrotic stromal context associated with local immune exclusion [121,122,123]. In digestive system tumors, current evidence more directly supports spatial and molecular associations among matrix-rich or high-stiffness stromal regions, altered TAM states, CAF proximity, fibrotic stromal remodeling, and barrier-like microenvironment formation. However, the TAM-intrinsic mechanotransduction mechanisms underlying these associations remain less directly defined in fibrotic septa of digestive tumors.

Evidence from broader tumor, fibrosis, biomaterial, and experimental systems suggests that LOX- and LOXL2-associated matrix crosslinking may promote collagen organization and stromal stiffening [124,125,126,127,128]; ITGB1-related matrix interaction may provide a possible link between macrophage or stromal-cell positioning and adhesion-dependent responses within collagen-enriched environments [126,127]; and FAK/PTK2-related signaling may connect matrix adhesion, mechanical sensing, and stromal-barrier formation [126,129,130]. Experimental studies further suggest that YAP/TAZ-, Piezo1–YAP-, or other mechanotransduction-related mechanisms may influence macrophage inflammatory responses, polarization states, stiffness sensing, or fibrosis-associated functions [124,125,129,130,131,132]. Therefore, in fibrotic septa of digestive system tumors, these pathways may help explain how matrix remodeling is linked to TAM adaptation, while their direct roles in TAM adaptation within this niche remain to be clarified by functional studies.

Functionally, fibrotic-septa TAMs are mainly associated with the preservation of a fibrotic, barrier-like stromal state and with local immune exclusion [65]. A persistently reinforced stromal barrier can restrict cell migration, tissue exchange, and local molecular diffusion, and may also weaken effector immune-cell entry and effective drug diffusion within tissues, thereby creating a microenvironment unfavorable for the full exertion of therapeutic effects [61,108,133]. Therefore, TAM adaptation to stromal constraint, immune exclusion, and restricted therapeutic response together constitute the major functional features of this niche.

### 3.4. TAM Features and Functions in Perivascular Regions

Perivascular regions place TAMs within an interface shaped by abnormal vascular architecture, perfusion fluctuation, and endothelial-derived signals. Because of their close spatial proximity to blood vessels or endothelial cells, these TAMs may participate in local microcirculatory states, vascular remodeling, and endothelial-interface regulation [65,73]. At the molecular level, perivascular TAM-associated programs commonly include angiogenesis- or vascular-remodeling-related factors such as VEGFA, ANGPT2, and MMP9 [134,135,136], together with recruitment- or positioning-associated molecules such as CXCR4 [137]. In addition, SPP1-related matrix remodeling and endothelial-interface regulatory features may also be involved, which may link these TAMs not only to angiogenic signaling but also to perivascular matrix remodeling and maintenance of abnormal vascular networks [138].

A key feature of this niche is that vascular proximity does not necessarily indicate a stable oxygen or nutrient supply. Tumor-associated vessels are often structurally abnormal and functionally unstable, and perivascular regions may therefore experience alternating blood flow, transient changes in vascular permeability, perfusion fluctuation, and local hypoxia. In this context, TAMs are better understood as cellular states responsive to fluctuations in local oxygen and nutrient availability [139]. At the same time, their location at the boundary between blood vessels and tumor parenchyma places them close to the routes of immune-cell recruitment, adhesion, and entry into tumor tissues, thereby allowing them to influence the spatial conditions for immune-cell entry [140].

The vascular background of different digestive system tumors further shapes the functional emphasis of perivascular TAMs. In liver cancer, the highly vascularized and sinusoidal architecture of the liver makes perivascular TAM states more closely linked to abnormal vascular remodeling, endothelial signaling, and immune-cell recruitment or retention. By contrast, in pancreatic cancer, perivascular localization often does not represent a stable supply zone because abnormal vessels coexist with stromal compression, insufficient effective perfusion, and microcirculatory instability [141,142]. Therefore, although vascular support and vascular remodeling constitute a shared axis of this niche, differences in the relative contribution of sinusoidal remodeling, abnormal angiogenesis, vessel compression, and perfusion instability across tumor contexts may further alter the functional emphasis of perivascular TAMs.

Functionally, perivascular TAMs connect abnormal vascular networks, microcirculatory states, and immune-entry conditions. Their angiogenesis- and remodeling-associated programs may contribute to vascular support, altered permeability, and maintenance of abnormal microcirculatory states, whereas their responses to perfusion fluctuation make them important regulatory nodes between the blood-flow system and tumor parenchyma. Meanwhile, their spatial proximity to endothelial interfaces may also allow them to alter local immune-infiltration patterns by shaping the conditions for immune-cell recruitment and entry [140]. Overall, perivascular TAMs mainly represent an interface-associated functional state organized around vascular remodeling, perfusion adaptation, and immune-entry regulation.

### 3.5. TAM Features and Functions in TLS-Adjacent Regions

TLS-adjacent regions are mainly shaped by dense local immune communication rather than by hypoxia, necrotic injury, or stromal-barrier pressure. In this niche, TAMs are often distributed at the edges of or around lymphocyte aggregates and remain in close spatial proximity to T cells, B cells, and dendritic cells, suggesting that their state formation is embedded within dense local immune-interaction networks [82,85,86]. Therefore, TLS-adjacent TAMs are better understood as plastic cellular states involved in immune organization and local immune-response regulation.

At the molecular level, TLS-adjacent TAMs commonly show features related to antigen presentation and immune communication, including MHC-II-related molecules such as HLA-DRA, HLA-DRB1, and CD74 [85,143,144]. In some contexts, they may also express inhibitory or checkpoint-associated molecules such as CD274 (PD-L1) and VSIR (VISTA), indicating that their molecular states can combine antigen-presentation capacity with immunoregulatory restraint [85,143,145]. This coexistence of antigen-presentation and immunoregulatory features gives TLS-adjacent TAMs substantial functional plasticity. When antigen presentation, T/B-cell interactions, and TLS-associated immune activation predominate, these TAMs may support local adaptive immune organization; when inhibitory signals and checkpoint restraint increase, they may also limit, balance, or reshape local immune responses [146,147].

The TLS architecture, lymphocyte composition, and immune background of different digestive system tumors further influence the functional orientation of TLS-adjacent TAMs. In colorectal cancer, these TAMs are more likely to be embedded in relatively active adaptive immune environments, where antigen-presentation and immune-communication programs can be linked to TLS-associated immune activation [86,148]. In liver cancer, chronic inflammation, hepatic immune tolerance, and therapy-induced changes in TLS morphology may more strongly shape their functional states, making them more likely to show bidirectional features in which immune activation and immune restriction coexist [85,149]. In gastric cancer, spatial analyses of TLS-associated immune landscapes also support the importance of macrophage or immune-cell interactions around TLSs, suggesting that their functions depend more on local immune organization than on a single macrophage phenotype [82,143].

Overall, TLS-adjacent TAMs occupy immune-dense interfaces characterized by the coexistence of antigen presentation, immune communication, and checkpoint-associated immunoregulation [82]. Whether they preferentially support immune activation or strengthen immune restriction depends on organ background, TLS maturation state, lymphocyte composition, cytokine balance, and the distribution of local inhibitory signals. Therefore, TLS-adjacent TAMs represent interface-associated states with bidirectional regulatory potential within TLS-associated immune networks.

### 3.6. TAM Features and Functions at Necrotic Borders

Necrotic borders form injury interfaces between necrotic tissue and adjacent viable tumor regions. In this niche, TAMs are continuously exposed to necrotic cell remnants, DAMPs, oxidative stress, and inflammatory mediators, and their states are therefore closely linked to injury-response, debris-clearance, and inflammatory-regulatory programs [92,93,150]. At the molecular level, inflammatory and inflammasome-related features may include IL1B, TNF, and NLRP3 [87,96], whereas damage-associated myeloid programs may involve S100A8 and S100A9 [36,151]. Debris-processing and protease-associated features such as CTSB further support the involvement of these TAMs in phagocytic clearance, tissue-remnant processing, and inflammatory signal amplification [36,152]. These programs are consistent with the concept that DAMP–inflammasome signaling can connect necrotic stimulation with myeloid-cell activation and tumor-promoting inflammatory responses [87].

The state of TAMs at necrotic borders is also shaped by metabolic disruption and tissue stress. Collapse of oxygen supply, accumulation of oxidative stress, and local metabolic imbalance may further amplify injury-response, debris-clearance, and inflammatory-regulatory programs in this region [92,100,153]. However, necrosis-associated inflammation does not necessarily represent effective antitumor immunity. Necrotic signals can enhance local inflammatory responses, but under persistent tumor-associated stress, these responses may gradually become coupled with immune restriction, repair-like tissue remodeling, and tumor-maintaining microenvironments. Thus, TAMs at necrotic borders often occupy mixed states in which inflammatory activation, debris clearance, stressed-tissue remodeling, and immunoregulatory restraint may coexist.

The functional meaning of necrotic borders depends on the tissue-injury background in which they are embedded. In liver cancer and some pancreatic cancers, necrosis, ischemia, and local inflammation are often intertwined, exposing TAMs to sustained injury stimulation within an evolving tumor environment. In lesions accompanied by chronic hepatitis or a fibrotic background, inflammatory activation and immunosuppression may further coexist or reinforce each other [95,96,154,155]. Therefore, necrotic-border TAMs are best interpreted in the combined context of acute damage-derived signals and chronic inflammatory or fibrotic backgrounds.

Overall, necrotic-border TAMs may participate in necrotic debris clearance, DAMP sensing, regulation of local inflammatory responses, and remodeling of stressed tissue interfaces; under persistent tumor-associated stress, these responses may also acquire immunoregulatory and repair-like features. These functions place necrotic-border TAMs at the intersection of injury clearance, inflammatory regulation, tissue remodeling, and immune restriction.

Across the niches discussed above, some representative TAM-associated molecules and programs recur in more than one spatial context. These recurrent features are better interpreted as niche-associated programs rather than niche-exclusive markers, because their functional emphasis depends on spatial location, co-expressed modules, neighboring-cell composition, and dominant local microenvironmental pressures. SPP1 is a representative example: SPP1-related features may be linked to remodeling or vascular adaptation in hypoxic or perivascular regions, whereas they may be more closely associated with tumor–stroma interface remodeling at the invasive front. This context-weighted interpretation helps explain why recurrent TAM-associated programs can appear across multiple niches while still contributing to distinct niche-associated functional states.

## 4. Cross-Niche Comparison: Major Functional Axes of TAM Spatial Heterogeneity

**Figure 2:**
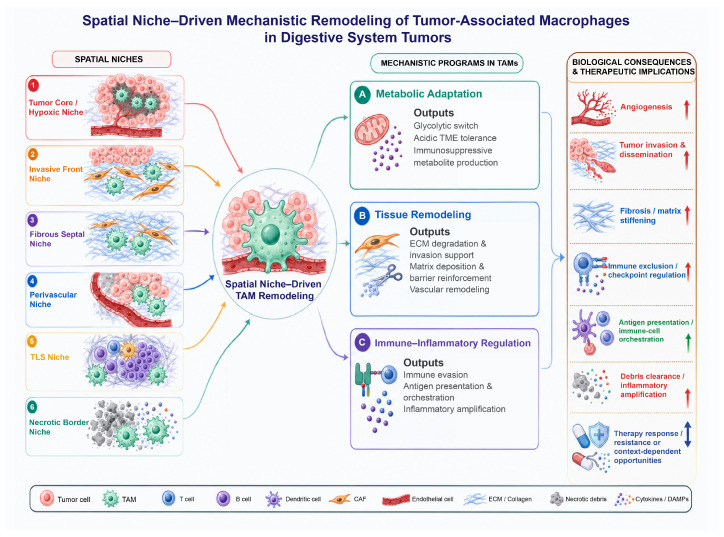
Spatial niche-driven functional remodeling of tumor-associated macrophages in digestive system tumors.

Although TAMs in different spatial niches exhibit distinct molecular features and functional activities, cross-niche comparison suggests that these differences arise from different combinations of shared programs rather than from fixed, isolated types. Local microenvironments determine where these programs are preferentially activated, how they are combined, and how they shape distinct functional emphases. As illustrated in Figure 2, these niche-shaped TAM states can be integrated into three cross-niche functional axes: metabolic adaptation, tissue remodeling, and immune-inflammatory regulation.

This schematic illustrates how six recurrent spatial niches may shape tumor-associated macrophage (TAM) remodeling and contribute to three cross-niche functional axes: metabolic adaptation, tissue remodeling, and immune-inflammatory regulation. The left panel summarizes the spatial niches considered in this review, including the tumor core/hypoxic niche, invasive front niche, fibrotic septal niche, perivascular niche, tertiary lymphoid structure-adjacent (TLS-adjacent) niche, and necrotic border niche. The middle panel organizes niche-shaped TAM remodeling into three functional programs and their representative outputs. The right panel summarizes major biological consequences and therapeutic implications, including angiogenesis, tumor invasion and dissemination, fibrosis and matrix stiffening, immune exclusion or checkpoint regulation, antigen presentation and immune-cell orchestration, debris clearance and inflammatory amplification, and context-dependent therapy response or resistance. Arrows indicate conceptual links among spatial niches, TAM functional programs, and biological or therapeutic outcomes. Abbreviations: TAMs, tumor-associated macrophages; CAFs, cancer-associated fibroblasts; ECM, extracellular matrix; TLS, tertiary lymphoid structure; DAMPs, damage-associated molecular patterns; DCs, dendritic cells; TME, tumor microenvironment.

### 4.1. Basic Nature of Cross-Niche Heterogeneity

Cross-niche TAM heterogeneity reflects the context-dependent combination of shared functional programs within distinct spatial microenvironments [156,157]. This heterogeneity appears as distinct state biases formed under different local conditions. These TAM state differences are usually continuous and context-dependent and are deeply embedded within local interaction networks composed of tumor cells, stromal cells, vascular structures, and other immune cells [17,158].

### 4.2. Three Functional Axes of Cross-Niche TAM Heterogeneity

Cross-niche comparison organizes TAM spatial heterogeneity around three intertwined functional axes: metabolic adaptation, tissue remodeling, and immune-inflammatory regulation. These axes represent recurrent functional directions that emerge across different local conditions, with each niche displaying distinct combinations and relative weights. Together, they provide an analytical structure for comparing recurrent TAM functional dimensions across spatial contexts. Spatial niches further indicate where these functions are preferentially activated, how they combine, and how local environments amplify specific functional programs.

Among these axes, metabolic adaptation reflects the continuous adjustments made by TAMs to maintain survival and function when local oxygen supply, nutrients, and material exchange are restricted. TAMs in the hypoxic core most prominently embody this direction, with their states mainly formed within local environments characterized by persistent hypoxia and metabolic constraint [100]. TAMs in perivascular regions more often reflect responses to perfusion fluctuations [73]. TAMs in fibrotic septa need to adapt to low-exchange environments caused by dense stroma and low permeability [159], whereas TAMs at necrotic borders also face metabolic disruption and oxidative stress accumulation [94]. Metabolic adaptation is therefore a shared TAM response to local oxygen, nutrient, and exchange constraints across multiple niches. It is often linked to pro-angiogenic and immunosuppressive programs and may help maintain abnormal oxygen supply and metabolic imbalance.

Tissue remodeling reflects the sustained rewriting of the extracellular matrix, tissue interfaces, vascular architecture, and post-injury local structures by TAMs. TAMs at the invasive front more prominently support ECM degradation, interface remodeling, and outward tumor expansion, whereas TAMs in fibrotic septa more prominently support fibrotic progression, stromal reinforcement, and barrier maintenance [160,161]. Meanwhile, pro-angiogenesis in the hypoxic core, vascular remodeling in perivascular regions, and tissue remodeling at necrotic borders also represent specific manifestations of this axis in different niches [55,73]. Tissue remodeling therefore spans several TAM-associated processes, including extracellular matrix turnover, interface reconstruction, stromal reinforcement, vascular remodeling, and post-injury structural rebuilding.

Immune-inflammatory regulation reflects the capacity of TAMs to regulate antigen presentation, immune-cell communication, immune restriction, inflammatory responses, and local post-injury reactions. TAMs in TLS-adjacent regions most directly embody this direction, with their roles concentrated in antigen presentation, support for T/B-cell interactions, and modulation of local immune restriction [82]. TAMs at necrotic borders more prominently exhibit inflammatory amplification, clearance of necrotic debris, and regulation of post-injury responses [93]. Meanwhile, immunosuppression in the hypoxic core, local immune restriction at the invasive front, and immune exclusion in fibrotic septa also indicate that this axis broadly participates in shaping local immune states across different niches [55]. Immune-inflammatory regulation is therefore regionally diversified across histological backgrounds. Spatial niches help distinguish its major facets, including antigen presentation, immune communication, immune restriction, inflammatory amplification, and injury response.

Overall, TAMs in different niches exhibit distinct weighted combinations of metabolic adaptation, tissue remodeling, and immune-inflammatory regulation. These axes are spatially intertwined and jointly shape the local functional states of TAMs. Their preferential niche distributions and cross-niche interpretations are summarized in Table 2. The three axes define major TAM functional dimensions, whereas spatial niches provide the histological context in which these functions are shaped and combined. In this framework, niche analysis helps clarify where TAM programs are activated and how they contribute to local microenvironmental states.

## 5. Therapeutic Implications and Perspectives

This section is intended as a hypothesis-generating discussion of spatially informed TAM-targeted intervention, rather than as a summary of clinically validated niche-specific therapeutic strategies. From this perspective, TAM-related therapeutic strategies may be refined not only by selecting specific targets, but also by considering local spatial context. Key considerations include which local environments should be prioritized, which dominant TAM states should be altered, and how these approaches might be combined with existing treatment modalities [57,144]. Accordingly, the following subsections discuss spatially informed intervention directions from three perspectives: niche-informed intervention, the strategic shift from broad depletion toward functional reprogramming, and the integration of spatial omics with functional validation.

### 5.1. Niche-Informed Intervention Directions

The spatial organization of TAM states provides a basis for prioritizing intervention directions according to dominant local pressures and functional programs. In hypoxic or poorly perfused regions, TAM programs linked to metabolic adaptation, angiogenesis, and immunosuppression support future testing of hypoxia- or metabolism-related modulation combined with TAM reprogramming [101,162,163]. In perivascular niches, the association of TAMs with abnormal vascular remodeling and immune-cell entry suggests potential combinations of vascular normalization, anti-angiogenic therapy, and macrophage reprogramming [138]. In invasive-front and fibrotic niches, TAM-related matrix remodeling and stromal-barrier maintenance support consideration of anti-fibrotic, anti-ECM-remodeling, or stromal-normalization approaches. Potential directions include targeting TAM–CAF communication or matrix crosslinking [164,165,166]. Around TLSs, TAM states associated with antigen presentation and checkpoint-linked immunoregulation suggest possible links between macrophage-focused immune reprogramming and immune checkpoint modulation [86,143]. At necrotic borders, injury-response and inflammatory-regulatory TAM states may inform combination strategies involving radiotherapy, chemotherapy, or innate immune stimulation, particularly in contexts involving antigen release and transient inflammatory responses [167].

Overall, niche-informed intervention analysis is best used to prioritize spatially defined functional programs for future testing. Further studies integrating spatial characterization, perturbation experiments, and therapeutic-response assessment will be needed to determine which TAM states are most actionable in specific digestive tumor contexts.

### 5.2. From “TAM Depletion” to “TAM Reprogramming”

Increasing evidence supports a view of TAMs as spatially dependent and functionally diverse cellular states shaped within different local environments [36,168]. This plasticity provides the rationale for therapeutic strategies that increasingly emphasize functional reprogramming based on spatial-niche features, rather than uniform TAM depletion [169]. Broad macrophage depletion may reduce selected tumor-promoting effects in the short term, but it may also impair antigen presentation, tissue clearance, and local homeostasis. This limitation is particularly relevant in niches where TAMs may show both immune-supportive and immunoregulatory activities [138,170,171]. A spatial-niche perspective therefore supports selective reprogramming strategies that alter dominant tumor-supportive TAM states while preserving or enhancing context-dependent immune-supportive functions. The shift from TAM depletion toward TAM reprogramming is best viewed as a strategic adjustment aligned with the spatial heterogeneity of TAMs in digestive system tumors. Linking TAM state transitions to therapeutic benefit within defined spatial contexts will be important for translating niche-guided reprogramming into more precise treatments.

### 5.3. Future Directions for Spatial Omics and Functional Validation

Future studies should further integrate spatial transcriptomics, multiplexed imaging, single-cell analysis, and functional experiments to link TAM localization with functional activity across different spatial niches. Existing studies have defined TAM distribution patterns, proximity relationships, and molecular states across different niches with increasing resolution, providing a basis for functional studies that test how TAMs contribute to immunoregulation, hypoxic adaptation, vascular support, and stromal remodeling within specific spatial contexts [172,173,174].

Methodologically, spatial localization information should be more closely integrated with interventional experimental designs. Spatial transcriptomics and multiplexed imaging can define TAM niches, neighboring-cell composition, and local signaling networks. Functional experiments, including single-cell state tracking, in vivo and in vitro coculture systems, organoid models, spatially directed perturbation, and lineage tracing, can further define how TAMs in specific niches contribute to immunoregulation, hypoxic adaptation, vascular support, and stromal remodeling. Representative niche-specific TAM-associated molecular programs, dominant biological roles, and evidence sources are summarized in Table 3 to clarify the evidence context for interpreting spatially defined TAM states and to support the prioritization of candidate programs for future functional validation. Based on the current evidence, niche-associated TAM programs can be interpreted with different levels of support. The hypoxic core, perivascular regions, and TLS-adjacent regions currently have relatively stronger support from digestive-tumor studies. Hypoxic-core TAMs are repeatedly linked to hypoxia- and metabolism-related programs, angiogenic signaling, and immunosuppressive features; perivascular TAMs are supported by evidence connecting them with angiogenesis, vascular remodeling, endothelial-interface regulation, and immune-cell entry conditions; and TLS-adjacent TAMs are increasingly linked to antigen-presentation programs, immune-cell communication, and checkpoint-associated immunoregulation. Fibrotic-septa TAMs have moderate support: their association with matrix-rich, stiff, and barrier-like stromal regions is supported, but TAM-intrinsic mechanotransduction programs and their direct roles in stromal-barrier maintenance remain less directly defined in digestive tumor tissues. Invasive-front and necrotic-border TAMs are supported mainly by spatial and molecular observations, whereas their functional roles are more inference-dependent. Invasive-front TAMs are associated with matrix degradation, stromal signaling, and tumor–stroma interface remodeling, but their direct contribution to invasion support or local immune restriction requires further functional testing. Necrotic-border TAMs are associated with damage-response, inflammatory, and debris-processing programs, whereas their causal roles in tissue remodeling, immune suppression, and therapy-response modulation remain less directly established. Future studies should therefore prioritize spatially resolved perturbation, lineage or state tracking, organoid and coculture systems, and therapeutic-response assessment to distinguish spatially enriched TAM states from functionally causal and therapeutically targetable programs.

## 6. Conclusions

In summary, TAM heterogeneity in digestive system tumors can be interpreted as dynamic states shaped by spatial niches and local microenvironmental pressures. Major spatial niches, including the hypoxic core, invasive front, fibrotic septa, perivascular regions, TLS-adjacent regions, and necrotic borders, provide a useful framework for organizing TAM-associated molecular features, functional tendencies, and potential intervention directions. By integrating dispersed spatial observations into a six-niche and three-axis framework, this review provides a conceptual basis for comparing TAM functional differentiation across local microenvironments and aligning niche-associated molecular programs with biological roles and evidence sources.

At the same time, the strength of evidence differs across niches and mechanisms. Some niche-associated TAM programs are supported by digestive-tumor single-cell, spatial, or mechanistic studies, whereas others remain based on broader tumor evidence, experimental models, or spatial and molecular associations that require further functional validation. These evidence boundaries highlight the prospective nature of the therapeutic strategies discussed here and position niche-guided TAM reprogramming as a promising direction for future spatially informed therapeutic exploration. Future studies should integrate spatial characterization, functional perturbation, lineage or state tracking, and therapeutic-response assessment. Such studies will help clarify the causal roles, applicable boundaries, and translational potential of niche-associated TAM programs in digestive system tumors.

## Figures and Tables

**Table 1:** Major spatial niches in digestive system tumors.

Spatial Niche	Defining Spatial Features	Dominant Local Pressures	Representative Digestive System Tumor Contexts
Hypoxic core	Viable deep intratumoral regions distant from effective perfusion, with restricted oxygen and nutrient exchange but without overt necrotic breakdown as the defining feature.	Persistent hypoxia; lactate accumulation; acidosis; nutrient deprivation; impaired perfusion; metabolic adaptation.	Pancreatic cancer; liver cancer; subsets of gastric cancer.
Invasive front	Dynamic tumor–stroma interface where tumor cells extend into surrounding tissue and remodel epithelial or stromal boundaries.	ECM remodeling; CAF activation; boundary disruption; invasive expansion.	Gastric cancer; colorectal cancer; liver cancer.
Fibrotic septa	Dense fibrotic bands or septa that compartmentalize tumor regions and restrict cellular and molecular transport.	Collagen deposition; matrix crosslinking; high stiffness; low permeability.	Pancreatic cancer; liver cancer; subsets of gastric cancer.
Perivascular regions	Regions surrounding abnormal tumor vasculature and endothelial interfaces that mediate exchange between circulation and tumor tissue.	Endothelial proximity; vascular permeability; perfusion fluctuations; vascular remodeling.	Liver cancer; pancreatic cancer; colorectal cancer.
TLS-adjacent regions	Regions around tertiary lymphoid structures enriched in T cells, B cells, dendritic cells, and antigen-presentation interactions.	Antigen presentation; lymphocyte communication; immune checkpoint signaling.	Colorectal cancer; gastric cancer; liver cancer.
Necrotic borders	Transition zones at the interface between necrotic foci and adjacent viable tumor tissue, enriched for necrotic debris and injury-response signals.	DAMP release; necrotic debris; phagocytic clearance; oxidative stress; inflammatory mediators; viable–necrotic interface.	Liver cancer; pancreatic cancer; advanced gastric or colorectal cancer.

CAFs, cancer-associated fibroblasts; DAMP, damage-associated molecular pattern; ECM, extracellular matrix; TLS, tertiary lymphoid structure.

**Table 2:** Major functional axes of TAM spatial heterogeneity across niches.

Functional Axis *	Niche Contexts Where Prominent	Cross-Niche Interpretation
Metabolic adaptation	Hypoxic core; perivascular regions; fibrotic septa; necrotic borders.	Adaptation to oxygen, perfusion, metabolic, and tissue-exchange constraints.
Tissue remodeling	Invasive front; fibrotic septa; perivascular regions; necrotic borders; hypoxic core.	Remodeling of matrix, tissue interfaces, vasculature, and injured structures.
Immune-inflammatory regulation	TLS-adjacent regions; necrotic borders; hypoxic core; invasive front; fibrotic septa.	Regulation of antigen presentation, immune communication, restriction, inflammation, and injury response.

* These functional axes should be interpreted as analytical dimensions rather than mutually exclusive categories. Each niche may combine multiple axes with different dominant functional weights. TLS, tertiary lymphoid structure.

**Table 3:** Niche-specific TAM-associated molecular programs, biological roles, and evidence sources.

Spatial Niche *	Representative TAM-Associated Molecules and Programs	Dominant Biological Role	Evidence Source
Hypoxic core	Hypoxia/metabolism: HIF1A, SLC2A1, LDHA [95,102,103]; angiogenesis/recruitment: VEGFA, CXCR4 [95,102,103]; immunosuppression: ARG1, CD163, MRC1 [101,105,106]; lactate-MCT-HIF signaling [101,106].	Metabolic adaptation; angiogenesis; immunosuppression.	Digestive-tumor evidence: Refs. [95,102,103,105].
Invasive front	ECM degradation/remodeling: MMP9, MMP14, CTSB, CTSD [111,112]; stromal signaling: TGFB1, SPP1 [111,113,114]; adhesion/matrix interaction: FN1, integrins [111,112].	Matrix degradation; interface remodeling; possible invasion support; possible local immune restriction.	Digestive-tumor evidence: Refs. [111,112,113,114].
Fibrotic septa	Matrix remodeling/stiffness-associated features: LOX, LOXL2 [126,127,128]; integrin-associated matrix interaction: ITGB1 [126,127]; FAK-related signaling [126,129,130].	Stromal barrier maintenance; matrix stiffening; immune exclusion; reduced drug penetration.	Digestive-tumor evidence: Refs. [121,126,127,128,175]; non-cancer/experimental evidence: Refs. [129,130].
Perivascular regions	Angiogenesis/vascular remodeling: VEGFA, ANGPT2, MMP9 [134,135,136]; recruitment/positioning: CXCR4 [137]; endothelial-TAM signaling [134,135,136,137].	Angiogenesis; vascular remodeling; perfusion adaptation; immune cell entry regulation.	Digestive-tumor evidence: Refs. [134,135,136,137].
TLS-adjacent regions	Antigen presentation: HLA-DRA, HLA-DRB1, CD74 [85,143,144]; checkpoint-associated regulation: CD274/PD-L1, VSIR/VISTA [85,143,145]; antigen-presentation and immune-checkpoint programs [85,143,144,145].	Antigen presentation; immune communication; checkpoint-linked regulation.	Digestive-tumor evidence: Refs. [85,143,144,145].
Necrotic borders	Inflammation/inflammasome: IL1B, TNF, NLRP3 [87,96]; damage-associated myeloid program: S100A8, S100A9 [36,151]; debris processing: CTSB [36,152]; DAMP-inflammasome signaling [87].	Damage response; debris clearance; inflammatory amplification; tissue remodeling; possible immune suppression.	Digestive-tumor evidence: Refs. [36,151,152]. Broader tumor evidence: Ref. [176].

* Molecules and pathways are representative programs or niche-associated features rather than niche-exclusive markers. Recurrent molecules such as SPP1 should be interpreted in relation to spatial location, co-expressed marker modules, neighboring-cell context, and dominant local pressures. Evidence source indicates the main literature context supporting each niche-associated mechanism. “Digestive-tumor evidence” refers to evidence from gastric, colorectal, liver, or pancreatic cancer. “Broader tumor evidence” refers to evidence from non-digestive tumor studies, whereas “non-cancer/experimental evidence” refers to non-cancer disease models, in vitro systems, biomaterial models, or other experimental settings. DAMP, damage-associated molecular pattern; ECM, extracellular matrix; FAK, focal adhesion kinase; MCT, monocarboxylate transporter; TAM, tumor-associated macrophage; TLS, tertiary lymphoid structure.

## Data Availability

No new data were created or analyzed in this study.
